# Effectiveness of Group Cognitive Behavioral Therapy and Exercise in the Management of Major Depressive Disorder: Protocol for a Pilot Randomized Controlled Trial

**DOI:** 10.2196/14309

**Published:** 2020-05-25

**Authors:** Mojtaba Yekrang Safakar, Marianne Hrabok, Liana Urichuk, Michal Juhas, Reham Shalaby, Devashree Parmar, Pierre Chue, Mark Snaterse, Judith Mason, Donna Tchida, Jill Kelland, Pamela Coulson, Daniella Sosdjan, Jason Brown, Katherine Hay, Deanna Lesage, Lacey Paulsen, Amy Delday, Sherianna Duiker, Shireen Surood, Adam Abba-Aji, Vincent Israel Opoku Agyapong

**Affiliations:** 1 Department of Psychiatry University of Alberta Edmonton, AB Canada; 2 Addiction and Mental Health, Alberta Health Services Edmonton, AB Canada; 3 Faculty of Social Work, University of Calgary Calgary, AB Canada; 4 Department of Psychiatry Faculty of Medicine and Dentistry University of Alberta Edmonton, AB Canada

**Keywords:** depression, major depressive disorder, cognitive behavioral therapy, group CBT, exercise

## Abstract

**Background:**

Despite evidence in scientific literature indicating the effectiveness of both cognitive behavioral therapy (CBT) and physical exercise in the management of major depressive disorder (MDD), few studies have directly compared them.

**Objective:**

This study aims to evaluate and compare the effectiveness of group CBT, physical exercise, and only wait-listing to receive treatment-as-usual (TAU) in the management of MDD. The investigators hypothesize that participants with MDD assigned to the group CBT or exercise arms of the study will achieve superior outcomes compared with participants wait-listed to receive TAU only.

**Methods:**

This prospective rater-blinded randomized controlled trial assesses the benefits of group CBT and exercise for participants with MDD. A total of 120 patients with MDD referred to addiction and mental health clinics in Edmonton, Canada, will be randomly assigned to one of the three equal-sized arms of the study to receive either weekly sessions of group CBT plus TAU, group exercise three times a week plus TAU, or only TAU for 14 weeks. Participants will be assessed at enrollment, 3 and 6 months post enrollment, midtreatment, and upon treatment completion for primary (functional and symptom variables) and secondary outcomes (service variables and health care utilization). In addition, participants in the intervention groups would be evaluated weekly with one functional measure. The data will be analyzed using repeated measures and effect size analyses, and correlational analyses will be completed between measures at each time point.

**Results:**

The study will be conducted in accordance with the Declaration of Helsinki (Hong Kong amendment) and Good Clinical Practice (Canadian guidelines). Written informed consent will be obtained from each subject. The study received ethical clearance from the Health Ethics Research Board of the University of Alberta on September 7, 2018 (Pro 00080975) and operational approval from the provincial health authority (Alberta Health Services 43638). As of October 13, 2019, we have enrolled 32 participants. The results will be disseminated at several levels, including patients, practitioners, academics, researchers, and health care organizations.

**Conclusions:**

The results of the pilot trial may inform the implementation of a multicenter clinical trial and provide useful information for administrators and clinicians who are interested in incorporating group CBT and group exercise interventions into existing care.

**Trial Registration:**

ClinicalTrials.gov NCT03731728; https://clinicaltrials.gov/ct2/show/NCT03731728

**International Registered Report Identifier (IRRID):**

PRR1-10.2196/14309

## Introduction

### Background and Rationale

Depressive disorders are a major public health problem. For example, the global prevalence of depressive disorders is over 4%, and depression is the single largest contributor to nonfatal health loss [1]. There is a need to identify interventions that are relatively cost-efficient, scalable, and can be offered to many people. Treatment for depressive disorders typically includes antidepressant medication and or counseling and psychotherapy.

Exercise as a form of treatment for depressive disorders, especially of mild-to-moderate severity, has evidence of benefit [2-5]. In fact, the magnitude of the effect of exercise as a treatment for depression is reported to be comparable with the magnitude of the effect of conventional treatment [6,7]. An umbrella review of systematic reviews and meta-analyses of the use of exercise to treat depressive symptoms in older adults, for example, concluded that “exercise is safe and efficacious in reducing depressive symptoms in older people” and that exercise “should be considered as a core intervention in the multidisciplinary treatment of older adults experiencing depression” [8]. A meta-analysis adjusting for publication bias concluded that “exercise has a large and significant antidepressant effect in people with depression” [9]. The mechanisms by which exercise decreases depressive symptoms may include biological mechanisms such as anti-inflammatory effects [10] or increasing neurotransmitter levels implicated in depression [11]. Other mechanisms may include increase in self-efficacy [10] or enhanced social interaction [12].

Despite this strong evidence base, few studies have incorporated multiple treatment conditions in a randomized controlled trial design, and few studies appear to have assessed the effect of group cognitive behavioral therapy (CBT) in comparison with exercise. A randomized clinical trial that assigned 54 mild-to-moderately depressed patients to a combined CBT plus exercise condition vs a CBT-only condition [13] found superior outcomes in suicidal ideation, depression, and activities of daily living in the combined condition group compared with the group receiving CBT only. However, few studies compared group CBT, group exercise, and wait-listing for treatment-as-usual (TAU) conditions. This is important in further delineating the effective components of treatment for mild-to-moderate depression, and the results have implications for service delivery and clinical recommendations in the treatment of depression within health care organizations in Alberta and beyond. Specifically, patients with major depressive disorder (MDD) referred to addiction and mental health clinics in Edmonton Zone may wait for weeks before receiving individual treatment. Thus, group treatment conditions examined in this study may serve as an expedient treatment avenue to decrease waiting list times for patients with MDD and improve outcomes.

### Aim and Objectives

The aim of this project is to conduct a randomized pilot trial to evaluate the feasibility and effectiveness of group CBT and group exercise on depressive symptom scores and functioning. The client outcomes will be organized according to functional variables (relationships, well-being, and physical activity), symptom variables (change in depressive symptoms scores), and service variables (patient compliance, retention in treatment, and patient satisfaction).

Given the aim, one objective of the project is to compare the mean changes in functioning, clinical symptoms, and service satisfaction variables from enrollment baseline to 12 weeks post enrollment for (1) participants receiving group CBT plus wait-listed to receive TAU, (2) participants receiving scheduled exercise plus wait-listed to receive TAU, and (3) participants only wait-listed to receive TAU. Another objective of the study is to compare the mean changes in functioning, clinical symptoms, and service satisfaction variables from treatment baseline to 7 and 14 weeks post treatment commencement for (1) participants receiving group CBT plus wait-listed to receive TAU and (2) participants receiving scheduled exercise plus wait-listed to receive TAU.

### Hypothesis

The investigators hypothesize that participants enrolled in the group CBT or group exercise treatments while wait-listed to receive TAU will achieve significantly lower symptom outcome measures scores at 12 weeks and 6 months post enrollment compared with participants only wait-listed to receive TAU on each outcome measure used. We expect that participants enrolled in the group CBT plus TAU arm will have outcomes comparable with those enrolled in the group exercise plus TAU arm of the study at 7 and 14 weeks post treatment commencement.

## Methods

### Overview of Study Design, Timeline, and Participant Selection

This study will be a longitudinal, prospective, parallel-design, three-arm, rater-blinded randomized clinical trial with a recruitment period of 6 months and an observation period of 14 weeks (plus waiting time) for each participant. The study will be conducted according to the timelines specified in the Gantt chart in Multimedia Appendix 1.

The research will be conducted in a municipal recreational center as well as addiction and mental health clinics in Edmonton, a large, sociodemographically diverse city in western Canada [14]. Potential participants will be recruited from the Addiction and Mental Health Intake Clinic in Edmonton. Patients with depression during the intake assessment who are presumed to meet the inclusion criteria of the study will be invited to enroll.

To confirm the diagnosis of MDD using Diagnostic and Statistical Manual of Mental Disorders, 5th Edition (DSM-5) criteria, potential participants will be sent to the mood and anxiety clinic or urgent clinics in Edmonton, where they will be assessed by a psychiatrist or psychiatry resident who is independent from the study team. The psychiatrist or resident may or may not initiate, continue, or adjust pharmacotherapy. The diagnosis will be communicated to the study coordinator and clinic nurse. Participants with MDD will be informed of their eligibility to participate in the study and will be considered for randomization after providing informed consent, whereas patients with other diagnoses will be informed of their exclusion from the study and will be directed to receive an appropriate treatment for their condition.

After the diagnostic confirmation by a psychiatrist, a research assistant who is trained in study procedures will provide the potential participants with an information leaflet about the study and answer any related questions they may have. All potential participants who agree to take part in the study will provide written informed consent before the completion of the baseline assessment measures and randomization.

Patients who are aged between 18 and 65 years, have been referred by a primary care provider or self-referred to the Addiction and Mental Health Intake Clinic in Edmonton, have received a primary diagnosis of MDD from a consultant psychiatrist based on DSM-5 criteria, and have provided written informed consent will be included in the study. Patients will be ineligible if they do not meet the above inclusion criteria; have not provided informed consent; or have a diagnosis of bipolar disorder, schizophrenia, or schizoaffective disorder.

At baseline, demographic and contact information will be collected. Participants’ name and contact information will be collected only for use in future communication or for the arrangement of treatment, assessment, and follow-up sessions.

Participants’ medical records will also be reviewed at two points, at enrollment and 6 months after enrollment in the study, to gather information about participants’ use of health services in the past 6 months to compare service utilization among the groups and to determine if participation in the intervention groups impacts the use of other health services in the short term. These data can also be used for any economic analyses (ie, cost-effectiveness) that will be conducted.

All the data will be stored for a minimum of 7 years before destruction as per the research ethics board’s requirements, and the research ethics board’s requirements pertaining to the collection and storage of information will be followed.

Multimedia Appendix 1 illustrates the Gantt chart for group CBT and exercise project.

### Interventions

Participants enrolled in the group CBT plus TAU condition will be wait-listed to receive TAU and will receive a 2-hour session of group CBT every week for 14 weeks. Participants enrolled in the group exercise plus TAU arm of the study will be wait-listed for TAU and will receive 60 min of scheduled and facilitated exercises three times a week for 14 weeks. Participants in the TAU-only arm of the study will be wait-listed to receive individual therapy or counseling from a therapist as per current standard protocol for managing patients with MDD in addiction and mental health clinics in Edmonton Zone. All above participants may or may not receive pharmacotherapy as prescribed by a psychiatrist who is independent from the study team.

#### Group Cognitive Behavioral Therapy

The group CBT will be offered at 3 addiction and mental health clinics in Edmonton: Edmonton Community Mental Health Clinic, Edmonton Hope and Wellness Centre, and Alberta Health Services (AHS) Clinical Psychology Service. All therapists will use a manualized CBT protocol with the same handouts and schedule developed based on the book *Mind Over Mood* [15]. The group CBT will be provided to a group of maximum 10 participants. Each session will be 2 hours long and will be conducted by certified therapists with special training to deliver group CBT. The structure of the session will be agenda setting, check-in, review of homework, new concepts or skills, homework assignment, and feedback.

#### Group Exercise

For scheduled group exercise, the research team will follow the current recommendations based on a literature review for the use of exercise for the treatment of depression and the Canadian Physical Activity Guide [16] recommendations. Scheduled group exercises incorporate the following parameters:

- Type: Aerobic or strength training exercises.
- Dose: Three times per week.
- Intensity: Moderate (participant’s self-rated physical activity of a 6 or 7 on the Borg Perceived Exertion Scale of 10 relative to the individual’s personal capacity). Moderate heart rate level will be calculated (65%-75% of maximum heart rate) for each participant at the beginning of the study, and participants will have access to heart rate monitors (worn on the wrist) to gain an understanding of what moderate intensity feels like.
- Time: 60 min in moderate heart rate zone per session, with three sessions per week (180 min per week).
- Duration: 14 weeks.
- Others with supervision: Physical exercise sessions will be run by CanFit Pro- or Alberta Fitness Leadership Certification Association–certified recreational therapists who will assess the safety of patients’ involvement in physical activity using the 2018 Physical Activity Readiness Questionnaire (PAR-Q+) before the initiation of the study and address any physical problems to minimize the risk of any adverse events happening during the sessions. The PAR-Q+ is a screening tool to determine safe participation in exercise. Participants identified as potentially at risk with physical activity will require clearance to participate by their medical doctor.

Participants will have an opportunity to choose from a variety of physical activities to facilitate a meaningful physical activity experience that is important for long-term maintenance. Participants will have the opportunity to engage in three of the following fitness programs per week for 14 weeks:

- Monday: individual fitness for 60 min
- Tuesday: group exercise or individual fitness for 60 min
- Wednesday: aqua or swimming for 60 min
- Thursday: track walking and group exercise for 60 min
- Friday: pole walking or hiking according to the season for 60 min.

All participants must consent to participate in the study-facilitated exercise groups to be considered in the study because supervision and guidance are required for safety and ensuring consistent results. During the trial, participants are encouraged to participate in the exercise options offered. If the participant engages in the exercise independently, it will be counted as one of their sessions. Once the trial is completed, they will be supported to continue with the exercise options in the community. Participants will be provided with fitness passes and equipment as needed. It will be explained to the participant that although the expense of the equipment is subsidized by the study team to facilitate their participation, they do not have to participate because of this supportive act. They may keep the equipment should they decide to withdraw from the study at any point.

For each session, 2 recreation therapists will provide programming to a maximum of 20 participants. During the sessions, the recreation therapists will provide participants with information regarding health, wellness, fitness training, and understanding exertion levels within exercise. Participants will be encouraged to participate at a moderate level of perceived exertion for the best results (6-7 on a 10-point scale). While engaging in the exercise programs, the participants will be self-reporting intensity using a rating of the perceived exertion scale to the facilitators. Each participant must attend at least 75% of programs (32 sessions out of 42 sessions) during the 14-week period to be considered as having completed the program and for data analysis purposes.

### Sample Size

As this is a pilot study, the research will utilize data that can be elicited from participants who can be enrolled within the existing operational resources and time frames. This method is acceptable for pilot studies with limited data on effect size and has been described by Haynes et al [17] as using “the patients I can get”. The study will therefore be limited to a sample size of 120, with about 40 patients recruited into each arm of the study.

### Outcome Measures

We will implement and evaluate the project using the Alberta Quality Matrix for Health [18].

The outcome measures are detailed in Multimedia Appendix 2. The primary outcomes include functional variables (relationships, well-being, and physical activity) and symptom variables (depression and risk). The secondary client outcomes include service variables (satisfaction and health utilization).

### Randomization and Blinding

Randomization will be enacted via randomly generated codes. Each study participant will receive a randomization code. As it will not be possible for participants to be blinded, treatment allocation will be made explicit to them as soon as randomization is concluded. The outcome assessors will be blinded to treatment group allocation by not involving them in discussions about the study participants and not granting them access to the database that contains the randomization code. In addition, study participants will self-complete all outcome assessments with the assessor facilitating procedural aspects if needed. Moreover, these assessors will not be involved in data analysis. After data collection is complete, all data will undergo a blind review for the purpose of finalizing the planned analysis.

### Follow-Up Assessment

Moreover, 12 and 24 weeks after baseline assessments, a blinded researcher will contact the study participants in all three arms of the study and assist them in the completion of a range of assessment tools related to the outcome measures. The number of treatment sessions (group CBT, group exercise, or individual therapy) received by the participants at each time point would be recorded for participants in all treatment arms. In addition, participants in the group CBT and exercise arms of the study would complete the assessment tools weekly during the sessions and also at midtreatment (7 weeks after starting treatment) and at the end of the treatment (14 weeks after the treatment commencement). These self-rated assessments would be coordinated by the group facilitators.

### Statistical Methods

The primary goal of the statistical analysis will be to produce summary descriptive statistics for the longitudinal data, which will provide estimates for future sample size calculations and enable calculation of effect size. For the three-arm trial, we will compare the mean change in scores for primary and secondary outcome measures from enrollment baseline to 12 weeks and 24 weeks post enrollment into the study, whereas for the two-arm trial, we will compare the mean change in scores for primary and secondary outcome measures from treatment baseline to 7 and 14 weeks post enrollment into treatment, in addition to comparing the trends in weekly change in scores on the Clinical Outcomes in Routine Evaluation-10-Outcome Measure between the 2 intervention groups. The data will be analyzed using repeated measures and effect size analyses, and correlational analyses will be completed between measures at each time point. The results of this study will guide the design for a future, more highly powered, study.

### Patient and Public Involvement

The study was designed and finalized based on the nonsystematic and informal feedback from the representative patients. This randomized trial also offers patients the opportunity to provide feedback via the patient satisfaction survey.

## Results

The study will be conducted in accordance with the Declaration of Helsinki (Hong Kong amendment) [1] and Good Clinical Practice (Canadian guidelines) [2]. Written informed consent will be obtained from each subject. The study has received ethical clearance from Health Ethics Research Board of the University of Alberta on September 7, 2018 (Pro 00080975) and operational approval from the provincial health authority, AHS, on September 12, 2018 (AHS 43638). The study is registered with ClinicalTrials.gov on October 21, 2018 (registration number NCT03731728). As of October 13, 2019, we had enrolled 32 participants. The results will be disseminated at several levels, including patients, practitioners, academics, researchers, and health care organizations.

The team of investigators will plan an organizational engagement strategy to advance discussions about feasibility and effectiveness before the conclusion of the trial. This will help ensure that the findings are a relevant part of the decision-making processes in a way that is aligned with the study findings as they emerge. This may facilitate the planning of a larger study that is endorsed at both leadership and operational levels so that the potential benefits of the interventions can reach patients in a more timely fashion.

## Discussion

### Expected Results

The results of the study will provide important information about the effectiveness of group CBT and or group exercise in the treatment of MDD. This will augment the literature in this area and also provide practical examination to see if benefits can be derived from the addition group treatment modalities to TAU. Currently, patients with MDD referred to addiction and mental health clinics in Edmonton Zone may wait for weeks before receiving care from a health care professional in a one-to-one setting. Long wait may negatively impact patients’ well-being, personal and occupational function, and satisfaction with care, and a group-based treatment may serve as an alternative for patients with MDD, which can be more expediently accessed.

The results of the pilot trial may inform the implementation of a multicenter clinical trial and provide useful information for administrators and clinicians who are interested in incorporating these interventions into existing care. The investigators expect that the pilot findings will inform and support administrative decision making with regard to further scaling and studying the intervention within the province of Alberta and beyond.

### Strengths of This Study

The following are the strengths of this study:

- Randomization of participants will ensure that patients in the three treatment arms have fairly similar psychiatric morbidity at baseline.
- Blinding of the outcome assessors and the use of self-rating scales for the primary outcome measures will ensure the elimination of bias in the outcome measures.

### Limitation of This Study

The rather small sample size may reduce the power of the study, which will limit the ability of the study to detect differences in outcome measures among participants in the three treatment arms.

## Appendix

Multimedia Appendix 1 Gantt chart for group cognitive behavioral therapy and exercise project.

Multimedia Appendix 2 Outcome measures.

## Abbreviations

AHS: Alberta Health Services
CBT: cognitive behavioral therapy
DSM-5: Diagnostic and Statistical Manual of Mental Disorders, 5th Edition
MDD: major depressive disorder
PAR-Q: Physical Activity Readiness Questionnaire
TAU: treatment-as-usual
