# Supplementary material for: Effectiveness of Group Cognitive Behavioral Therapy and Exercise in the Management of Major Depressive Disorder: Protocol for a Pilot Randomized Controlled Trial
Source: JMIR Res Protoc. 2020 May 25;9(5):e14309. doi: 10.2196/14309 (PMC7281203; doi:10.2196/14309)
Supplement: Multimedia Appendix 1 [file resprot_v9i5e14309_app1.docx]

|  |  | **Year 1** | | | | Year 2 | |
| --- | --- | --- | --- | --- | --- | --- | --- |
|  |  | **Start Date-End Date** | | | | **Start Date-End Date** | |
| **Milestone No.** | **Milestones** | **Q1** | **Q2** | **Q3** | **Q4** | **Q1** | **Q2** |
| **Milestone 1:**  **Setting up of infrastructure for group CBT and gymnasium** | | | | | | | |
| **1.1** | Assembling of group CBT facilitators and setting of gymnasium in the community |  |  |  |  |  |  |
| **Milestone 2:**  **The recruitment of study participants** | | | | | | | |
| **2.1** | Recruitment, baseline assessment, randomization |  |  |  |  |  |  |
| **2.2** | Assignment into one of the three arms of the study |  |  |  |  |  |  |
| **2.3** | Delivery of group CBT, exercise, and/or TAU to participants |  |  |  |  |  |  |
| **Milestone 3:**  **Follow-up assessment of study participants** | | | | | | | |
| **3.1** | Follow-up assessments of individual study participants (Excluding satisfaction surveys) |  |  |  |  |  |  |
| **3.2** | Follow-up satisfaction survey of participants all groups |  |  |  |  |  |  |
| **Milestone 4: Data compilation, analysis and preparation of reports, publications and presentations** | | | | | | | |
| **4.1** | Data compilation |  |  |  |  |  |  |
| **4.2** | Data Analysis |  |  |  |  |  |  |
| **4.3** | Preparation of reports, publications and presentations |  |  |  |  |  |  |
