# Supplementary material for: Effectiveness of Group Cognitive Behavioral Therapy and Exercise in the Management of Major Depressive Disorder: Protocol for a Pilot Randomized Controlled Trial
Source: JMIR Res Protoc. 2020 May 25;9(5):e14309. doi: 10.2196/14309 (PMC7281203; doi:10.2196/14309)
Supplement: Multimedia Appendix 2 [file resprot_v9i5e14309_app2.docx]

|  | Construct | Tool | Rater | Time Required | Time Points Assessed | | | | | | |
| --- | --- | --- | --- | --- | --- | --- | --- | --- | --- | --- | --- |
|  |  |  |  |  | At Enrollment | At  Start of group CBT or Exercise | 12 weeks  Post enrolment | Weekly^a^ during group CBT or Exercise | 7weeks after start of  group CBT or Exercise | 14weeks  after start of  group CBT or Exercise | 6 months after enrollment |
| Symptom Variables | Depressive symptoms | Beck Depression Inventory (BDI-II) | Client | 5 min | X | X | X |  | X | X | X |
|  | Symptoms, risk level | CORE OM (Symptoms and Risk subscales) | Client |  | X | X | X |  | X | X | X |
| Functional variables | Inter- personal functioning | CORE OM (Functioning subscale) | Client | 10 min for 34-items of CORE OM |  |  |  |  |  |  |  |
|  | Well-being | CORE OM (Well-being subscale) | Client |  |  |  |  |  |  |  |  |
|  | Physical Activity | International Physical Activity Questionnaire | Client | 5 min | X | X |  |  | X | X |  |
| Symptom and functional variables | Symptoms, risk, interpersonal functioning and wellbeing | CORE-10 OM | Client | 5 min |  |  |  | X |  |  |  |
| Service variables | Patient Satisfaction with Service | Addiction and Mental Health Client Experience Survey | Client | 5 min |  |  |  |  | X | X |  |
|  | Health Utilization | Data extraction | Research Team | - | X |  |  |  |  |  | X |
|  |  | Total Time for participant | |  |  | 20 min |  |  | 25 min | 25 min |  |
|  |  |  |  |  |  |  |  |  |  |  |  |
| ^a^Except week 7 and 14 when CORE 34 OM will be administered | | | | | | | | | | | |
